# Bionic smart recycled paper endowed with amphiphobic, photochromic, and UV rewritable properties†

**DOI:** 10.1039/d0na00627k

**Published:** 2020-08-25

**Authors:** Guofeng Zhang, Guopeng Chen, Fuchao Yang, Zhiguang Guo

**Affiliations:** Ministry of Education Key Laboratory for the Green Preparation and Application of Functional Materials, School of Materials Science & Engineering, Hubei Key Laboratory of Polymer Materials, Hubei University Wuhan 430062 China yfc@hubu.edu.cn; State Key Laboratory of Solid Lubrication, Lanzhou Institute of Chemical Physics, Chinese Academy of Sciences Lanzhou 730000 People's Republic of China zguo@licp.cas.cn

## Abstract

The single-use of large volumes of paper has become a serious issue which is depleting our resources and damaging the environment. It is of great significance and challenging to adopt simple, reasonable and practical methods to prepare functional recyclable paper. In this article, inspired by pleochromatic creatures and plant leaves' special wettability, a series of photochromic amphiphobic recycled paper (PAR_*i*_) products was successfully prepared by adding gourd-like modified tungsten trioxide (MTT) to waste paper pulp. The results show that PAR_2–7_ has excellent lyophobic performance and amazing photochromic properties. It is worth noting that PAR_7_ has an impressive amphiphobic behavior, and its surface water contact angle (WCA) and oil contact angle (OCA) are 146 ± 1° and 137 ± 1°, respectively. It can withstand continuous ultraviolet light irradiation for 60 h, indicating excellent resistance to ultraviolet radiation. Most importantly, the reversible photochromic properties of PAR_7_ make it possible to write repeatedly on the surface by using ultraviolet light. In short, the performance of the prepared PAR is stable and superior, which can not only alleviate paper waste, but also means it has great potential in the fields of decoration, packaging, and banknote anti-counterfeiting technology.

## Abstract 2

Photochromic amphiphobic recycled paper (PAR) has hydrophobic, oleophobic, photochromic and UV repeated writing properties.

## Graphical abstract

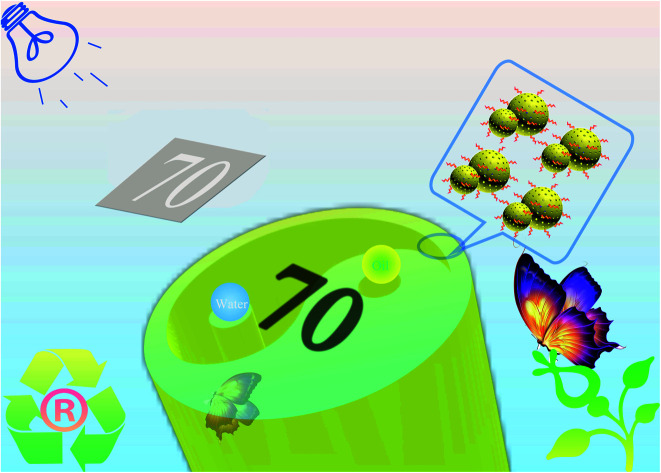

## 1. Introduction

Biological surfaces have evolved unique structures and performances to combat against harsh environments or for the purposes of reproduction. For example, the color of butterfly wings changes based on their special microstructures.^1,2^ In real life, smart materials with color-changing properties have great application potential in anti-counterfeiting and information display technology. Allochroic paper which can be written on with UV light would be quite interesting. Also, paper is a medium for writing and printing, which provides a material basis for the inheritance of culture. Log pulp is a fibrous substance obtained by processing grass and trees and is a necessity for making cultural paper. In addition, cultural paper can also be produced by replacing waste wood pulp with waste paper, which is called recycled paper. At present, the main advantages of recycled paper being widely studied and prepared are listed as follows:^3–5^ (1) it can promote the environmental protection and stability of paper manufacturing; (2) it can slow down the rate of logging to avoid the destruction of ecosystems by human activity; (3) it can make paper multi-functional and expand its applications. Therefore, recycled paper is the best way to maintain the relative balance of the ecosystem and promote the recycling of paper. Unfortunately, ordinary recycled paper and traditional paper are susceptible to liquids in real life,^6^ and even a small number of wrinkles after drying will affect the aesthetic properties of the paper. Therefore, recycled paper with a smart way of writing and anti-wetting properties is highly desirable.

Papermaking is a great invention from ancient China. Generally speaking, we add various types of secondary components in the papermaking process to increase the beauty and texture of the paper.^7–10^ For example, Si *et al.* modified stearic acid with dopamine and magnesium hydroxide and successfully prepared super-hydrophobic recycled paper with flame retardant properties.^11^ Wen *et al.*^12^ successfully mixed cotton cellulose and stearate to prepare a colored superhydrophobic paper with uniform internal and external wettability. Yang *et al.*^13^ successfully prepared magnetic superhydrophobic flame retardant composite paper with ultra-long hydroxyapatite nanowires combined with magnetic nanoparticles. Yang *et al.* fabricated superhydrophobic paper with a photothermal stimulation response, which could be driven by controlled light.^6^ Therefore, we believe that adding a suitable secondary component to recycled paper can not only achieve an anti-wetting effect but can also help to improve the performances of recycled paper.

The secondary component we chose is tungsten trioxide (TT). It is an indirect semiconductor with photochromic properties^14,15^ and is commonly used to prepare catalysts, coatings, and fireproof materials.^16–20^ So far, artificially prepared TT and other smart semiconductors have been reported to show a variety of morphologies such as nanowires, nanotubes, nanoflakes, nanospheres, nanosheets, coral-like nanostructures, *etc.*^16,21–25^ Their main preparation methods include the hydrothermal method,^14,21^ solvothermal method,^26,27^ template-assisted method,^24^*etc.* However, these methods have the disadvantages of long cycles, complicated processes, high temperatures, and high pressures, which make the preparation cost extremely high. Herein, the chemical precipitation method we proposed can not only overcome the aforementioned shortcomings but also has the advantage of a high yield, which is much preferred by researchers. Furthermore, we expect to load TT prepared by a precipitation method into the pulp to produce a novel type of recycled paper that is not limited to a single performance compared to other impressive reports of multifunctionality.^22^

In this work, we first purposefully picked a plant leaf (*Chenopodium album* L. (CAL)) that grew in the field, and our test shows that it has excellent superhydrophobicity and a multi-level micro–nano structure. Inspired by its hierarchical structure and wettability, we have developed a new type of functionalized recycled paper with photochromism, hydrophobicity, and oleophobicity. The gourd-like tungsten trioxide was modified with fluorosilane and then introduced into waste paper pulp. The PAR was successfully prepared by a simple vacuum suction filtration process. The prepared PAR paper has an excellent processing performance, stable wettability, and photochromism. It can absorb ultraviolet light to force the internal tungsten trioxide to undergo a valence state change, which is reflected as a reversible color change macroscopically. In short, the prepared PAR not only plays an important role in alleviating paper waste but also has broad application prospects in the fields of anti-counterfeiting technology, decoration and packaging.

## 2. Experimental

### 2.1 Materials

The waste paper was printing paper used in the laboratory. Calcium chloride, citric acid, sodium tungstate, ethanol, nitric acid, sodium hydroxide, and 1*H*,1*H*,2*H*,2*H*-perfluorooctyltriethoxysilane (PFOS) were purchased from Sinopharm Chemical Reagent Co., Ltd. Rapeseed oil, soybean oil, arowana oil, and motorcycle oil were all purchased from the local market. All the above experimental chemicals were not purified further before use.

### 2.2 Preparation of tungsten trioxide (TT)

**Scheme 1:**
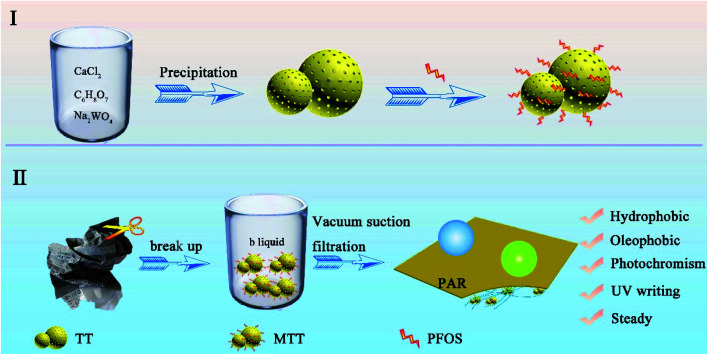
The preparation process of the TT microsphere (I) and PAR samples (II).

Part I in Scheme 1 shows the process of preparing TT.^28^ First, calcium chloride (0.027 mol) was dissolved in 60 mL of deionized water and citric acid (0.001 mol) was dissolved in 20 mL of deionized water. After mixing, 160 mL deionized water was added and magnetically stirred for 10 minutes to form a homogeneous solution (denoted as liquid A). Sodium tungstate (0.303 mol) was dissolved in 60 mL of deionized water and slowly added dropwise to liquid A. Next, the sodium hydroxide solution was added to adjust the pH to 12, while continuing to stir for 30 min. Then, it was washed three times with deionized water and ethanol, dried, and the white precipitate was collected. Finally, the white precipitate was immersed in a mixed solution of nitric acid (*c* = 3.02 mol L^−1^) until its color turned yellow, and the final product was TT.

### 2.3 Modification of tungsten trioxide

The WO_3_ product was soaked in a PFOS (0.8 mL)/ethanol (30 mL) solution. This self-assembly process lasted for 12 h under magnetic stirring. Then, the upper liquid was collected and denoted liquid B. The sediment was washed, dried and collected to obtain modified tungsten trioxide (MTT).

### 2.4 Preparation of PAR

Part II of Scheme 1 shows the preparation process of PAR. First, a piece of waste printing paper (weight 5 g) was cut it into small fractions and soaked in deionized water (200 mL). Then the temperature was raised to 90 °C and stirred for at least 3 h to form the fiber pulp. Then, the pH value was adjusted to 9 utilizing sodium hydroxide, and the pulp cake was obtained by suction filtration through a circulating water vacuum pump (SHZ-DIII). Secondly, the dry pulp cake (0.5 g) was added into a mixed solution containing ethanol (30 m) and deionized water (30 mL) and stirred for 2 h at 80 °C to disperse it again. Adjusting the temperature to 50 °C, different amounts of MTT (0 g, 0.05 g, 0.1 g, 0.2 g, 0.3 g, 0.4 g, and 0.5 g) and 20 mL of ethanol were added to the pulp, stirred for 30 min and named samples 1–7. In addition, liquid B (5 mL) was added to samples 2–6. Finally, after vacuum suction filtration, mechanical tableting (HY-12 Infrared Tablet Press) and drying, the series PAR_*i*_ was successfully prepared (i takes a value of 1–7, indicating the different MTT contents from 0–0.5 g).

### 2.5 Characterization and analysis methods

Field emission scanning electron microscopy (FESEM, Sigma 500) with energy dispersion spectroscopy (EDS) was used to characterize the samples’ morphology and element composition distribution. An X-ray diffractometer (D8 Advance) was used to characterize the crystal structure and composition of the samples. X-ray photoelectron spectroscopy (Escalab 250xi) and Fourier infrared spectrometry (Nicolet is50) were employed to analyze the molecular structure and chemical composition of the samples. The Raman spectrum of the sample was obtained by a Raman spectrometer (LabRAM HR Evolution) using a 532 nm wavelength excitation source. Under air atmosphere, a thermogravimetric analyzer (TGA 1) characterized the weightlessness of the samples at a heating rate of 10 °C min^−1^. The average water contact angles (WCAs) and oil contact angles (OCAs) were obtained by measuring five different positions on the sample surface at room temperature using a contact angle measuring instrument (JC 2000D1) (droplet volume ∼ 5 μL). The distance between the UV lamp (JT8-Y 15 W, central wavelength 253.7 nm and rated power 15 W) and the sample was set at 5 cm to test the photochromic properties and writability of the sample. In the mechanical stability test, the PAR_7_ samples were face down on the abrasive paper for the metallograph (1500 meshes). PAR_7_ samples under 294 N in the normal direction were moved 10 cm away by an external drawing force and the test was repeated at this amplitude. The WCA and OCA were measured firstly after 20 cm wear length and then every 40 cm wear length.

## 3. Result and discussion

### 3.1 Biological inspiration

**Fig. 1:**
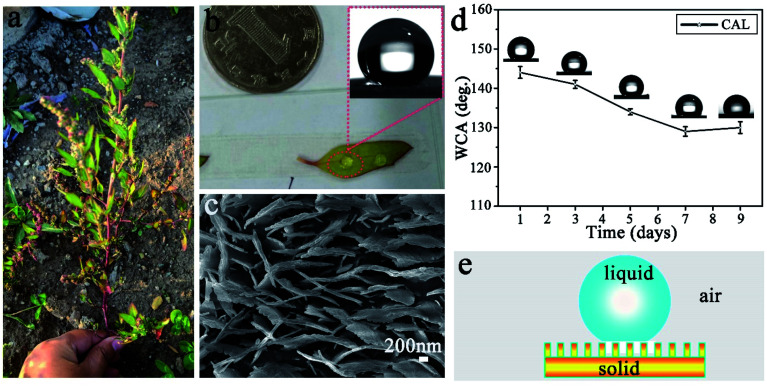
(a) An optical photo of the plant CAL. (b) An optical picture of water droplets on the surface of the blade. The illustration is a contact angle picture (WCA = 151 ± 2°). (c) SEM image of the blade surface. (d) At room temperature, the relationship between the WCA of the CAL blade surface and the placement time. (e) The Cassie model for the liquid contact interface.

Fig. 1a shows a photograph of CAL found in Changchun City, China. It is about 40 cm high, the stem is thick and purple-red, and the leaves have an oval surface with a very thin white component (wax). Water droplets are ellipsoidal and easily roll off the surface of freshly picked leaves (Fig. 1b) with excellent super-hydrophobic properties (Fig. 1b, inset). Fig. 1c is a SEM image of the CAL surface. It is clear that there are many irregular polygons connected on the CAL surface (ESI, Fig. S1a†). A large number of lamellae are stacked upright in each polygon, and there are gaps between adjacent lamellae (ESI Fig. S1b†). There is a toe-like structure at the top, with an average toe width of 50–60 nm and a height of 20–30 nm (Fig. 1c). The Cassie model can be used to explain its excellent superhydrophobic properties.^29^ Water droplets are in a discrete three-phase equilibrium state (solid phase, liquid phase, and gas phase) on the solid surface as shown in Fig. 1e. Once the balance is broken, the air cushion will reduce the resistance, causing the water droplets to roll down quickly. However, the super-hydrophobicity of the CAL surface is not stable. When the blade is alone in the air, the WCA will quickly decrease and show poor durability (Fig. 1d). It is the micro-nano structure and wax content of the CAL surface that make the water droplets have a high contact angle value and easily roll-off.^30,31^ Therefore, inspired by the hierarchical structure and wettability of CAL, we prepared a gourd-like MTT with a micro-nano structure.

### 3.2 Performance and characterization of TT and MTT

**Fig. 2:**
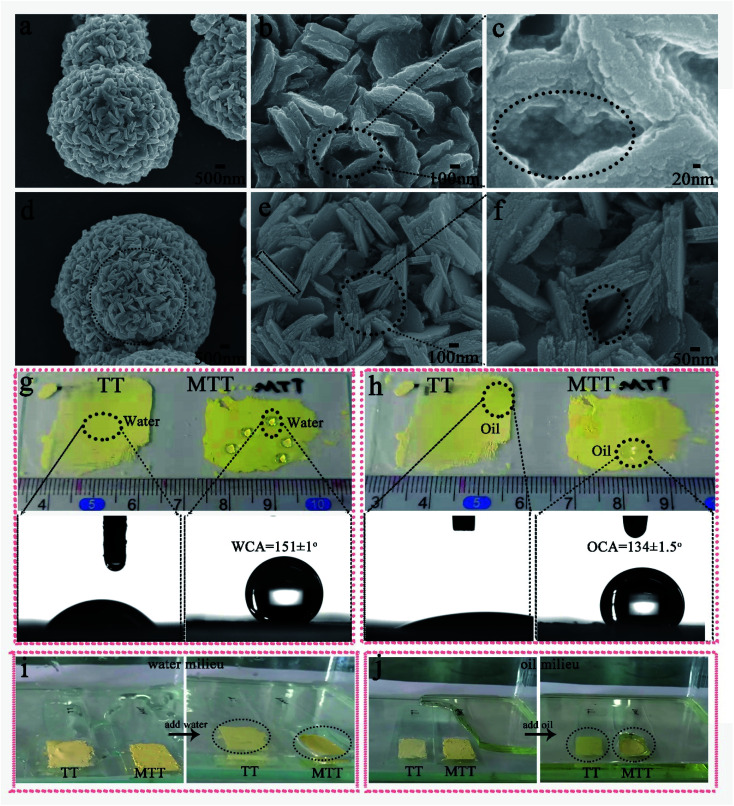
FESEM images of TT (a–c) and MTT (d–f). The profiles and WCAs of water droplets (g) and rapeseed oil droplets (h) on the surfaces of TT and MTT. The TT and MTT placed in water (i) and rapeseed oil (j) milieux to observe wettability differences intuitively.

We first prepared gourd-like TT particles by the precipitation method according to Scheme 1 part I, and then modified them by utilizing fluorosilane to obtain the MTT ingredient by a one-pot method. Fig. 2a–c and d–f are the FESEM images of TT and MTT, respectively. The morphology of both was gourd-like composed of small balls (diameter ∼ 6 μm) and large balls (diameter ∼ 10 μm). In addition, the assemblage of each sphere is composed of a large number of sheets (long ∼ 600 nm and wide ∼ 100 nm) and a void-like structure. The similar morphology of these two samples shows that the one-pot modification process has little effect on the surface morphology of TT. Fig. 2g and h show the profiles of water and rapeseed oil droplets on the surface of yellow powder TT and MTT, respectively. When the droplets (water and oil) fall on the TT surface, they spread quickly, but on the MTT surface, they gather in an elliptical shape with a high contact angle value (WCA = 151 ± 1°, OCA = 134 ± 1.5°). This phenomenon macroscopically proved that the gourd-like tungsten trioxide was successfully modified. To intuitively illustrate the wettability difference, TT and MTT were glued to the glass slide and placed in water (Fig. 2i) or rapeseed oil (Fig. 2j) milieu at the same time. The results show that the MTT surface can resist the water and rapeseed oil covering to some extent, and form a distinct liquid pit while that of TT does not exist.

**Fig. 3:**
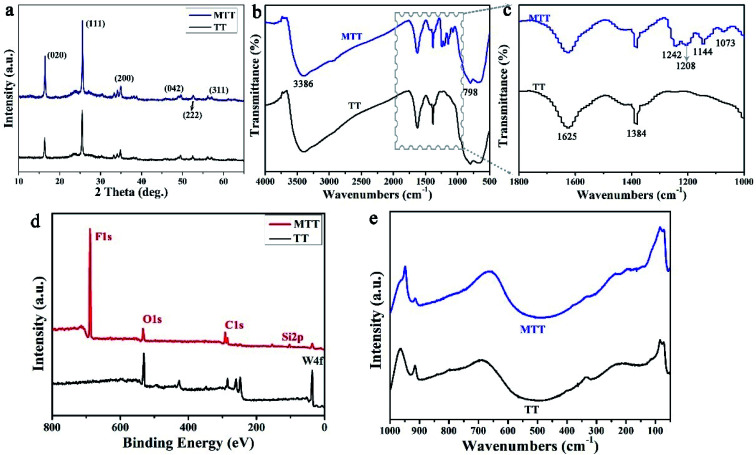
The XRD patterns (a), FT-IR spectra (b and c), XPS spectra (d) and Raman spectra (e) of the TT and MTT samples.

For a deeper level of research, we conducted component analysis on TT and MTT. As shown in Fig. 3a, the XRD pattern of TT and MTT indicates that the 2*θ* positions of the spectral peaks are consistent, appearing at about 16°, 25°, 35°, 49°, 53°, and 57°. Thus, the samples are orthogonal phase WO_3_·H_2_O.^28^ This result can also be obtained from Raman spectroscopy (Fig. 3e). Obviously, the peaks located at 686.6 cm^−1^, 914.8 cm^−1^, and 964.1 cm^−1^ correspond to the O–W–O, W–O, and W=O vibration modes.^32,33^ Also, as shown in Fig. 3b and c, the infrared spectra of TT and MTT have the same vibration peaks at 3386 cm^−1^ (structured water), 1625 cm^−1^ (symmetrical OH tensile and bending vibration), 1384 cm^−1^ and 798 cm^−1^ (W–O–W).^34–37^ In addition, the vibration peaks of MTT at 1208 cm^−1^ (C–F_3_ group) and 1073 cm^−1^ (Si–O vibration) indicate that the chemical modification was successful.^35,38^ As shown in Fig. 3d, the XPS spectra of TT and MTT have the same peaks at 286 eV, 530 eV, and 38 eV, corresponding to the C1s, O1s, and W4f, respectively. In addition, MTT has obvious F1s and Si1s peaks at 688.6 eV and 103.4 eV. The presence of the F and Si elements is not only good evidence for the successful modification of TT, but also the reason why it avoids being wetted by liquids.^39,40^ In short, we prepared gourd-like tungsten trioxide and successfully modified it.

### 3.3 PAR performance and application

**Fig. 4:**
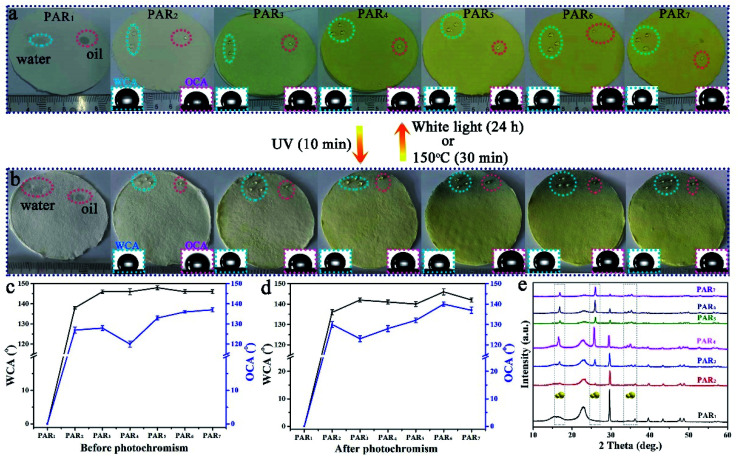
(a) Optical pictures of PAR_*i*_ before photochromism; (b) optical pictures of PAR_*i*_ after photochromism. The illustrations correspond to the contact angle pictures of water and oil. The blue dashed circles are water and the red dashed circles are rapeseed oil. The statistics of the WCA and OCA of PAR_*i*_ before photochromism (c) and after photochromism (d). (e) XRD patterns of PAR_*i*_.

We have successfully produced different types of PAR_*i*_ through Scheme 1 Part II. As shown in Fig. 4a, the recycled paper has the same size (diameter ∼ 7 cm and thickness ∼ 1 mm), and the color gradually deepens with the increased amount of MTT (white → yellow), which indicates that MTT and waste paper pulp is very well mixed during the preparation process. PAR_1_ is easily wetted by water (or oil) due to the absence of MTT, showing the original color of the pulp (white). As for the specimens of PAR_2–7_, although the loading amounts of MTT are different, the water and rapeseed oil droplets almost bead up on the PAR_2–7_ recycled paper surfaces (Fig. 4a, inset). In addition, when the recycled paper is irradiated with ultraviolet light for about 10 minutes, it will gradually change to tungsten cyan (Fig. 4b); conversely, when the discolored recycled paper is placed under white light for about 24 hours or heated at 150 °C for about 30 minutes, the original color will be restored afterward. It is worth noting that PAR_1_ has no color change during this process, which indicates that the cause of the color change of recycled paper is not pulp. Fig. 4c and d are the contact angle statistics of recycled paper before and after photochromism, respectively. Obviously, the WCA and OCA of the recycled paper did not change much before and after the photochromic process, which shows that the ultraviolet light irradiation has little effect on the wettability of the recycled paper. As shown in Fig. 4e, the XRD pattern of PAR_1_ shows that the peaks at 2*θ* = 15.2° and 23.4° belong to natural cellulose.^41,42^ The XRD patterns of PAR_2–7_ have peaks of natural cellulose and MTT, indicating that MTT and cellulose were successfully mixed. Besides, the recycled paper has a certain flame retardancy due to the addition of MTT particles. After PAR_1_ was burned, the ashes were grayish-white, indicating complete combustion. The color of the PAR_2–7_ ashes gradually increased with the increase of MTT, indicating insufficient combustion (ESI, Fig. S2†).

**Fig. 5:**
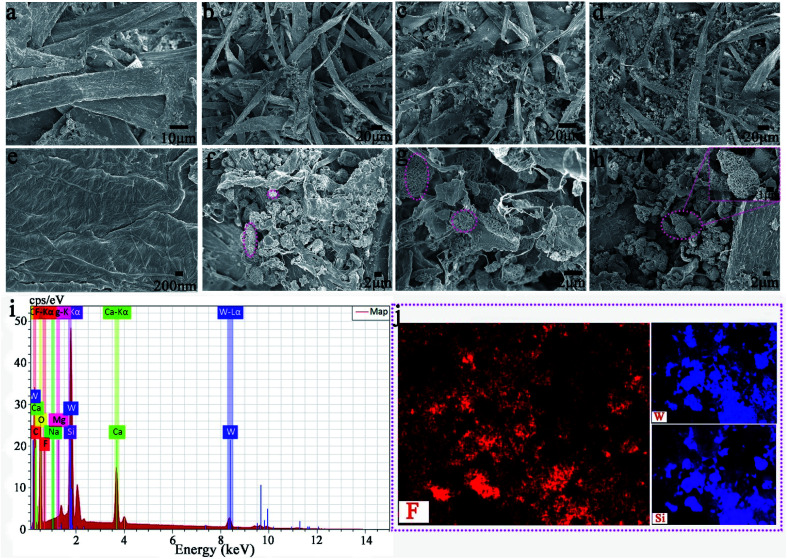
(a–d) and (e–h) are the low magnification SEM and high magnification SEM of PAR_1_, PAR_3_, PAR_5_, and PAR_7_, respectively. EDS spectrum (i) and some elemental mapping (j) of PAR_7_.

Fig. 5a–h shows the FESEM images of recycled paper (PAR_1_, PAR_3_, PAR_5_ and PAR_7_). A large number of fibers in PAR_1_ are intertwined (Fig. 5a), and the surface is relatively smooth (Fig. 5e). EDS analysis shows that PAR_1_ mainly contains C, O, Na, Si, Ca, and Mg elements (ESI, Fig. S3a†), and is evenly distributed on the surface (ESI, Fig. S3b†). The aggregation degree of MTT in waste paper pulp further reflects the difference in the initial addition amount. From PAR_2_ to PAR_7_, the aggregation degree of the particles gradually increases (Fig. 5b–d). It is worth noting that the aggregation degree of the particles is the largest in PAR_7_, and you can see “gourd” particles mixed with fibers (Fig. 5h). This indicates that PAR_7_ was successfully loaded onto the gourd-shaped MTT. Fig. 5i and j are the EDS and element mapping of PAR_7_. PAR_7_ not only contains all the elements of PAR_1_ (ESI, Fig. S3b†) but also the Si element content is significantly higher than PAR_1_ (Fig. 5i). This is mainly due to the addition of liquid B and MTT during the preparation process. In addition, PAR_7_ contains a large amount of uniformly distributed elements F and Si (Fig. 5j), which is one of the reasons for its high contact angle values.^43,44^ So we finally selected PAR_7_ with a relatively superior performance for subsequent stability characterization and research.

**Fig. 6:**
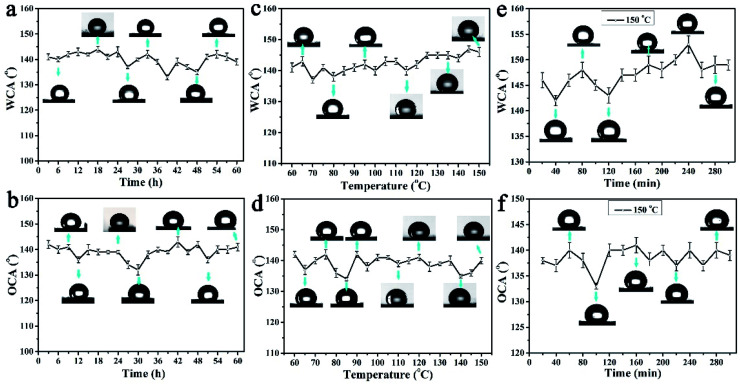
Statistical graph of the WCA (a) and OCA (b) on the surface of PAR_7_ under different ultraviolet irradiation times. The distance between the UV light source (15 W) and the recycled paper is about 5 cm. Statistical graphs of WCA (c) and OCA (d) with different temperatures on the surface of PAR_7_. From 60 °C to 150 °C, the temperature was increased by increments of 5 °C, and PAR_7_ was placed at each temperature for 30 min. The change of WCA (e) and OCA (f) on the surface of PAR_7_ with heating time at 150 °C.

Due to the tungsten trioxide ingredient, the colour of the PAR_7_ surface will change greatly under ultraviolet light irradiation. To clarify the robustness of the amphiphobic properties, we tested the WCA and OCA stability of the PAR_7_ recycled paper surface through designing multiple types of experiments below. Fig. 6a and b show the changing trend of the static WCA and OCA on the surface of PAR_7_ under different illumination times with ultraviolet irradiation. The sample surfaces' WCA and OCA remained stable during the entire 60 hours of UV irradiation, which indicates that PAR_7_ has excellent anti-UV stability. Fig. 6c and d are the statistical graphs of the changes of WCA and OCA on the surface of PAR_7_ with various temperatures. Obviously, the WCA will slightly increase with increasing temperature. WCA reaches its maximum when the temperature reaches 145 °C. The value of OCA has a large variation range in the range of 60–90 °C, it is relatively stable between 90–150 °C, and the maximum value is 141 ± 1° at 150 °C. PAR_7_ has good thermal stability with almost no decomposition below 300 °C (ESI, Fig. S4†). Therefore, based on the WCA and OCA of PAR_7_ at different temperatures, we chose 150 °C as the best follow-up research temperature. As shown in Fig. 6e and f, the WCA and OCA of the PAR_7_ surfaces heated at 150 °C for different times are provided. Overall, PAR_7_ has the highest WCA and OCA when heated at 150 °C for 4 hours. Besides, the mechanical abrasion tests show that the lyophobicity of PAR_7_ remained stable after a relative sandpaper wear length of 180 cm. When the wear distance is greater than 180 cm, the CA shows a slow and continuous downward trend (ESI, Fig. S5†). Most importantly, PAR_7_ is also universal for soybean oil (OCA = 138 ± 1°), arowana oil (OCA = 144 ± 2°) and motorcycle oil (OCA = 140 ± 2°) (ESI, Fig. S6†).

### 3.4 PAR_7_ surface UV writing and mechanism analysis

**Fig. 7:**
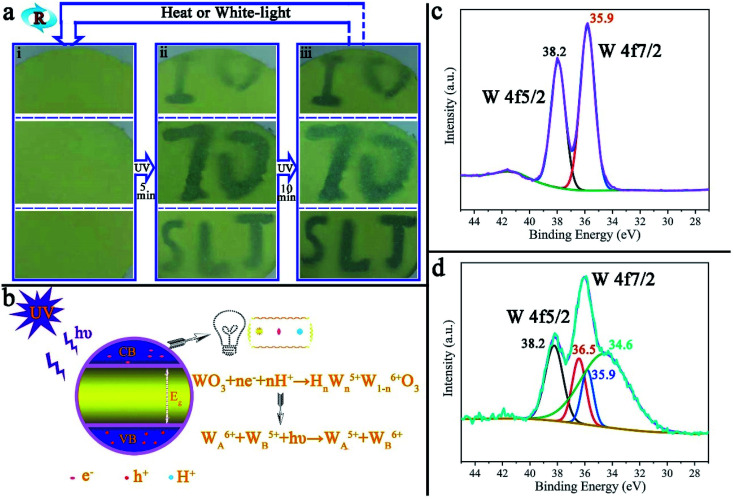
(a) UV light repeated writing on the surface of PAR_7_ and (b) the PAR_7_ photochromic principle diagram. The XPS pattern of the W element in MTT before (c) and after (d) photochromism.

Photochromism is one of the most promising characteristics of tungsten trioxide. Fig. 7a is reversible repetitive writing by ultraviolet light on the surface of PAR_7_. With the aid of the mold (ESI, Fig. S7†), blue characters will gradually appear on the surface of PAR_7_ with ultraviolet light irradiation and deepen accordingly. When the surface of PAR_7_ is irradiated under white light (or heated), the characters will disappear. The important thing is that this reversible UV writing can be repeated at least 10 times, which mainly benefits from the MTT in the recycled paper. Fig. 7b is the principle diagram for MTT photochromism. When the photon energy of ultraviolet light (*hυ*) is greater than the band-gap energy (*E*_g_) of MTT, it will excite the electrons in the valence band (VB) to overcome the energy barrier level and transition to the conduction band (CB), making the VB generate photogenerated electrons (e^−^) and holes (h^+^). h^+^ has strong oxidizing properties and can degrade moisture in the air into H^+^. Then, e^−^ and H^+^ further react with WO_3_ on the body surface to form tungsten bronze. Therefore, the color change of WO_3_ is mainly due to the charge transfer between W^5+^ and W^6+^.^45,46^ The photochromic mechanism of tungsten trioxide can be further verified by XPS. As shown in Fig. 7c, before MTT discoloration, W4f peaks appeared at 38.2 eV and 35.9 eV, corresponding to W4f5/2 and W4f7/2, respectively, indicating that the valence state of tungsten on the sample surface was +6.^28,47^ However, after photochromism, the W4f pattern showed a total of four peaks, two located at 38.2 eV and 35.9 eV, corresponding to the binding energy values of W^6+^, and two at 36.5 eV and 34.6 eV, corresponding to the binding energy values of W^5+^, as shown in Fig. 7d.^28,47,48^ This indicates that the valence state transition of the W element exists on the sample surface, which further verifies the rational explanation of the photochromism.

## 4. Conclusions

Recycled paper not only has a long life cycle but can also be multifunctionally modified by adding a secondary component. In this work, we successfully prepared functionalized PAR_*i*_ inspired by the CAL structure and wettability. First of all, a gourd-like TT powder was quickly prepared by chemical precipitation. Secondly, the TT was successfully modified by a one-pot method to obtain MTT (WCA = 151 ± 1° and OCA = 134 ± 1.5°). The similar micro-topography of TT and MTT has different surface wettability, which shows that the chemical composition has a significant effect on it. Finally, MTT was loaded into the waste paper pulp, and then a new multi-functional PAR was successfully developed through vacuum suction filtration. The prepared PAR_7_ has a highly stable surface hydrophobicity (WCA = 146 ± 1° and OCA = 137 ± 1°) and reversible photochromic properties. The WCA and OCA on the surface of PAR_7_ can be continuously irradiated by ultraviolet light for 60 h to maintain stability. More importantly, the prepared PAR_7_ has reversible photochromic properties and its surface can be repeatedly written on by ultraviolet light through a simple design. Therefore, the prepared recycled paper is expected to be used in decoration, ultraviolet detection of factory leaks, anti-counterfeiting technology and other related fields.^45^

## Conflicts of interest

There are no conflicts to declare.

## Supplementary Material

NA-002-D0NA00627K-s001

## References

[cit1] Han Z., Yang M., Li B., Mu Z., Niu S., Zhang J., Yang X. (2016). J. Bionic Eng..

[cit2] Niu S., Li B., Ye J., Mu Z., Zhang J., Liu Y., Han Z. (2016). Sci. China: Technol. Sci..

[cit3] Dexter M., Rickman K., Pan C., Chang C.-h., Malhotra R. (2019). J. Cleaner Prod..

[cit4] Liu Y., Shen W., Man Y., Liu Z., Seferlis P. (2019). Comput. Ind. Eng..

[cit5] Corcelli F., Fiorentino G., Vehmas J., Ulgiati S. (2018). J. Cleaner Prod..

[cit6] Yang R.-L., Zhu Y.-J., Chen F.-F., Qin D.-D., Xiong Z.-C. (2018). ACS Sustainable Chem. Eng..

[cit7] Balea A., Sanchez-Salvador J. L., Monte M. C., Merayo N., Negro C., Blanco A. (2019). Molecules.

[cit8] Schnell C. N., Tarrés Q., Galván M. V., Mocchiutti P., Delgado-Aguilar M., Zanuttini M. A., Mutjé P. (2018). Cellulose.

[cit9] Di J., Sun Q., Song X. (2018). Carbohydr. Polym..

[cit10] Tarres Q., Oliver-Ortega H., Alcala M., Merayo N., Balea A., Blanco A., Mutje P., Delgado-Aguilar M. (2018). Carbohydr. Polym..

[cit11] Si Y., Guo Z. (2016). J. Colloid Interface Sci..

[cit12] Wen Q., Guo F., Yang F., Guo Z. (2017). J. Colloid Interface Sci..

[cit13] Yang R.-L., Zhu Y.-J., Chen F.-F., Qin D.-D., Xiong Z.-C. (2019). ACS Sustainable Chem. Eng..

[cit14] Huo X., Zhang H., Shen W., Miao X., Zhang M., Guo M. (2019). J. Mater. Chem. A.

[cit15] Zhang J., Fu X., Hao H., Gan W. (2018). J. Alloys Compd..

[cit16] Liu T., Zhang X., Huang T., Yu A. (2018). Nanoscale.

[cit17] Hasani A., Le Q. V., Tekalgne M., Guo W., Hong S. H., Choi K. S., Lee T. H., Jang H. W., Kim S. Y. (2018). ACS Appl. Mater. Interfaces.

[cit18] Xie S., Chen Y., Bi Z., Jia S., Guo X., Gao X., Li X. (2019). Chem. Eng. J..

[cit19] Xiao X., Zhou X., Ma J., Zhu Y., Cheng X., Luo W., Deng Y. (2019). ACS Appl. Mater. Interfaces.

[cit20] Guo H., Jiang N., Wang H., Shang K., Lu N., Li J., Wu Y. (2019). Chemosphere.

[cit21] Pan J., Wang Y., Zheng R., Wang M., Wan Z., Jia C., Weng X., Xie J., Deng L. (2019). J. Mater. Chem. A.

[cit22] Ge B., Han L., Liang X., Li F., Pu X., Zhu X., Zhang Z., Shao X., Jin C., Li W. (2018). Appl. Surf. Sci..

[cit23] Li K., Shao Y., Yan H., Lu Z., Griffith K. J., Yan J., Wang G., Fan H., Lu J., Huang W., Bao B., Liu X., Hou C., Zhang Q., Li Y., Yu J., Wang H. (2018). Nat. Commun..

[cit24] Herdt T., Deckenbach D., Bruns M., Schneider J. J. (2019). Nanoscale.

[cit25] Sun Z., Huo R., Choi C., Hong S., Wu T.-S., Qiu J., Yan C., Han Z., Liu Y., Soo Y.-L., Jung Y. (2019). Nano Energy.

[cit26] Zhang D., Fan Y., Li G., Ma Z., Wang X., Cheng Z., Xu J. (2019). Sens. Actuators, B.

[cit27] Pu W., Song Z., Yan J., Xu H., Ji H., Yuan S., Li H. (2019). J. Mater. Sci..

[cit28] Jiang T., Guo Z. (2016). Appl. Surf. Sci..

[cit29] Cassie A. B. D., Baxter S. (1944). Trans. Faraday Soc..

[cit30] Feng L., Li S., Li Y., Li H., Zhang L., Zhai J., Song Y., Liu B., Jiang L., Zhu D. (2002). Adv. Mater..

[cit31] Barthlott W., Neinhuis C. (1997). Planta.

[cit32] Yang F., Wang F., Guo Z. (2018). J. Colloid Interface Sci..

[cit33] Acharyya S. S., Ghosh S., Bal R. (2015). Chem. Commun..

[cit34] Li Q., Guo Z. (2019). J. Colloid Interface Sci..

[cit35] Li Q., Guo Z. (2019). Nanoscale.

[cit36] Evdokimova O. L., Kusova T. V., Ivanova O. S., Shcherbakov A. B., Yorov K. E., Baranchikov A. E., Agafonov A. V., Ivanov V. K. (2019). Cellulose.

[cit37] Khan A., Bhosale N. Y., Mali S. S., Hong C. K., Kadam A. V. (2020). J. Colloid Interface Sci..

[cit38] Zuo K., Wu J., Chen S., Ji X., Wu W. (2019). Cellulose.

[cit39] Sun Y., Guo Z. (2019). Nanoscale.

[cit40] Wang X., Zeng J., Yu X., Zhang Y. (2019). J. Mater. Chem. A.

[cit41] Fu J., Yang F., Guo Z. (2019). Mater. Lett..

[cit42] Vasconcelos N. F., Feitosa J. P. A., Andrade F. K., Miranda M. A. R., Sasaki J. M., Morais J. P. S., e Silva L. M. A., Canuto K. M., Rosa M. d. F. (2019). Cellulose.

[cit43] Zhao X., Wei J., Li B., Li S., Tian N., Jing L., Zhang J. (2020). J. Colloid Interface Sci..

[cit44] Liu K.-F., Li P.-P., Zhang Y.-P., Liu P.-F., Cui C.-X., Wang J.-C., Li X. J., Qu L. B. (2019). Cellulose.

[cit45] Hui B., Wu D., Huang Q., Cai L., Li G., Li J., Zhao G. (2015). RSC Adv..

[cit46] Adachi K., Mita T., Tanaka S., Honda K., Yamazaki S., Nakayama M., Goto T., Watarai H. (2012). RSC Adv..

[cit47] Wei J., Jiao X., Wang T., Chen D. (2015). J. Mater. Chem. C.

[cit48] Ling Z., Liu K., Zou Q., Li Q., Zhang K., Cui Z., Yuan W., Liu Y. (2018). RSC Adv..

