# Supplementary material for: Bionic smart recycled paper endowed with amphiphobic, photochromic, and UV rewritable properties
Source: Nanoscale Adv. 2020 Aug 25;2(10):4813–21. doi: 10.1039/d0na00627k (PMC9419801; doi:10.1039/d0na00627k)
Supplement: NA-002-D0NA00627K-s001 [file NA-002-D0NA00627K-s001.pdf]

Electronic Supplementary Material (ESI) for Nanoscale Advances.
This journal is © The Royal Society of Chemistry 2020

Supplementary Information

Bionic smart recycled paper endowed with amphiphobic, photochromic, repeatedly UV writable utility

Guofeng Zhang,[a] Guopeng Chen [a], Fuchao Yang [a*] and Zhiguang Guo[*ab]

[a]Hubei Collaborative Innovation Centre for Advanced Organic Chemical Materials and Ministry of Education Key Laboratory for the Green Preparation and Application of Functional Materials, Hubei University, Wuhan 430062, People's Republic of China.

[b]State Key Laboratory of Solid Lubrication, Lanzhou Institute of Chemical Physics, Chinese Academy of Sciences, Lanzhou 730000, People's Republic of China.

*Corresponding author. Email: Yang's Email: yfc@hubu.edu.cn.; Guo's Email: zguo@licp.cas.cn.

Supplementary Figures

Figure S1. (a-b) FESEM images of Chenopodium album L. leaves.

Figure S2. (a) $PAR_{1\text{-}7}$ (long ~ 4.5 cm, width ~ 1.5cm) (b) The tweezers clamped $PAR_{1\text{-}7}$ and ignited in the flame lasting for 3 s, then moved away from the embers that allowed it to burn naturally in the air.

Figure S3. EDS (a) and element mapping (b) of PAR_1. (c) Element mapping of C, O, Ca, Na, and Mg in PAR_7.

Figure S4. Thermogravimetry curves of PAR_1 and PAR_7 samples.

Figure S5 (a) The illustration for mechanical wear test. The WCA (b) and OCA (c) of PAR_7 after different wear length. The PAR_7 surface of was loaded with 294 N (normal direction) and then grind it horizontally with sandpaper (1500 meshes).

Figure S6. The OCA of soybean oil (a), motorcycle oil (b), and arowana oil (c) on the surface of PAR_7.

Figure S7. Homemade simple mold: (a) I♡-shape; (b) 70-shape; (c) SLJ-shape.

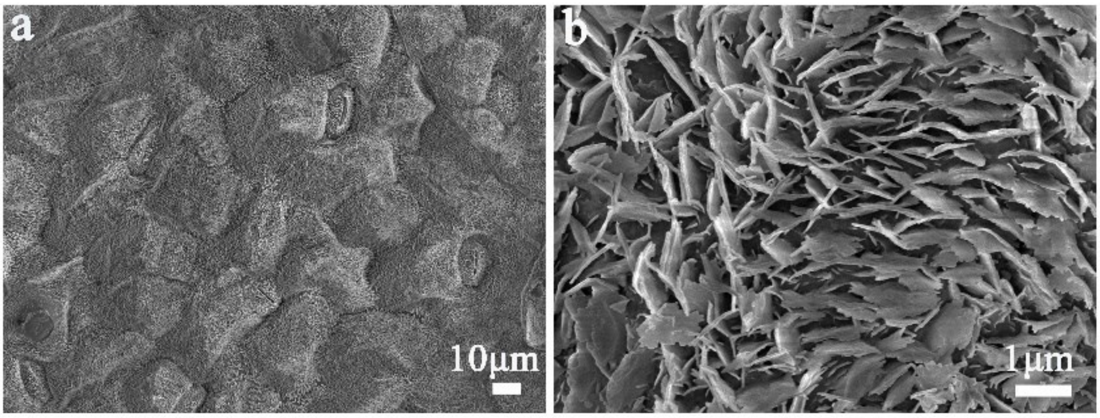

Figure S1 (a-b) FESEM images of Chenopodium album L. leaves.

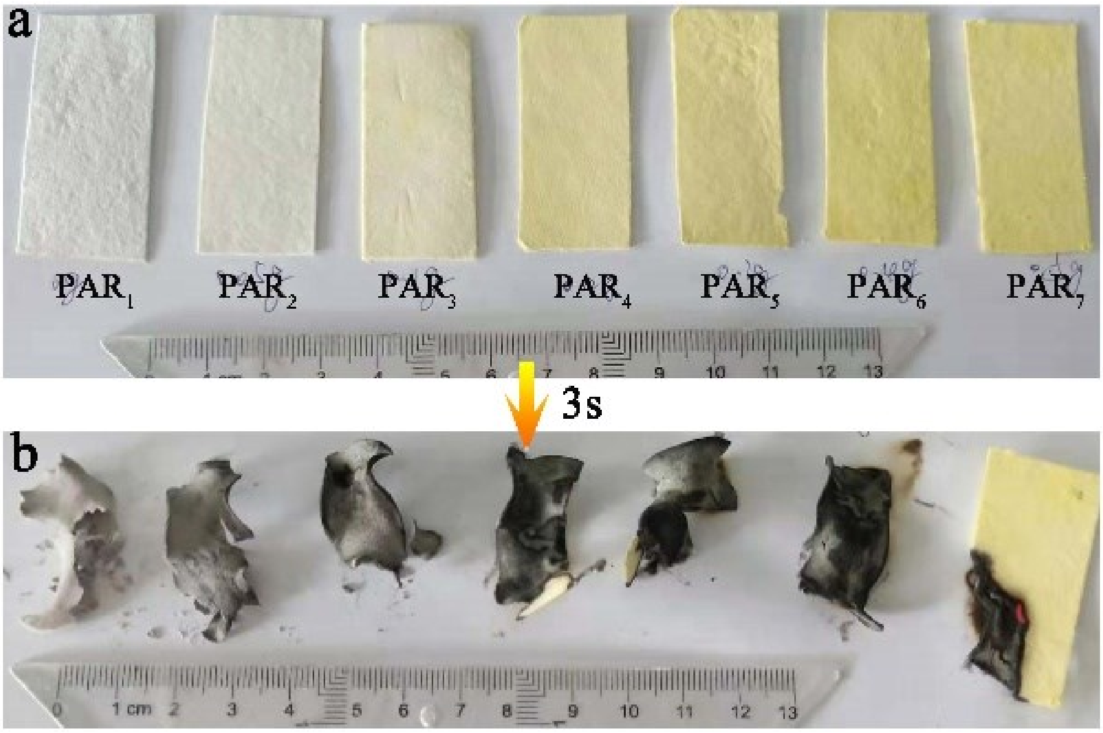

Figure S2 (a) PAR_{1-7} (long ~ 4.5 cm, width ~ 1.5cm) (b) The tweezers clamped PAR_{1-7} and ignited in the flame of the flame for 3 s, then moved away from the embers that allowed it to burn naturally in the air.

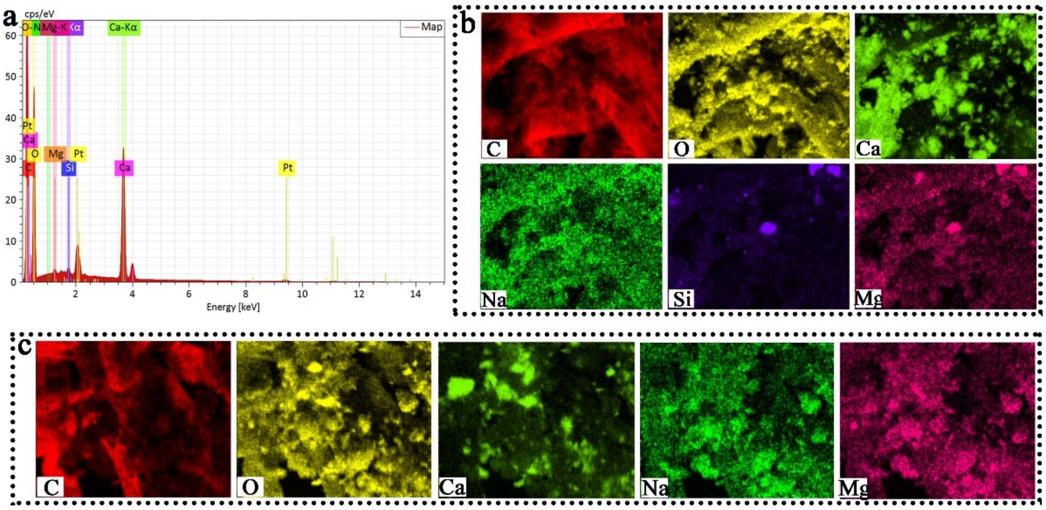

Figure S3 EDS (a) and element mapping (b) of PAR_1. (c) Element mapping of C, O, Ca, Na, and Mg in PAR_7.

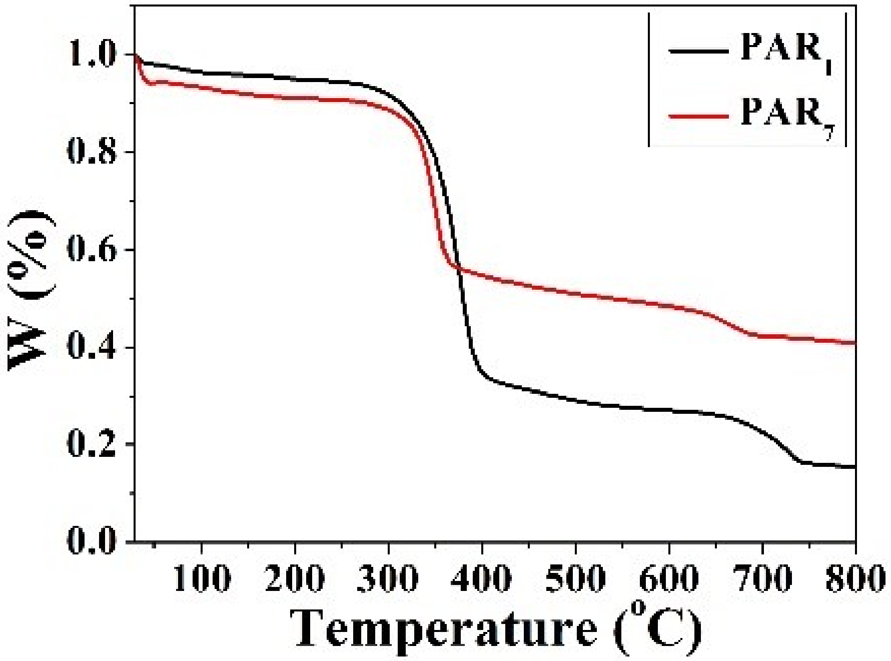

Figure S4 Thermogravimetry curves of PAR_1 and PAR_7 samples.

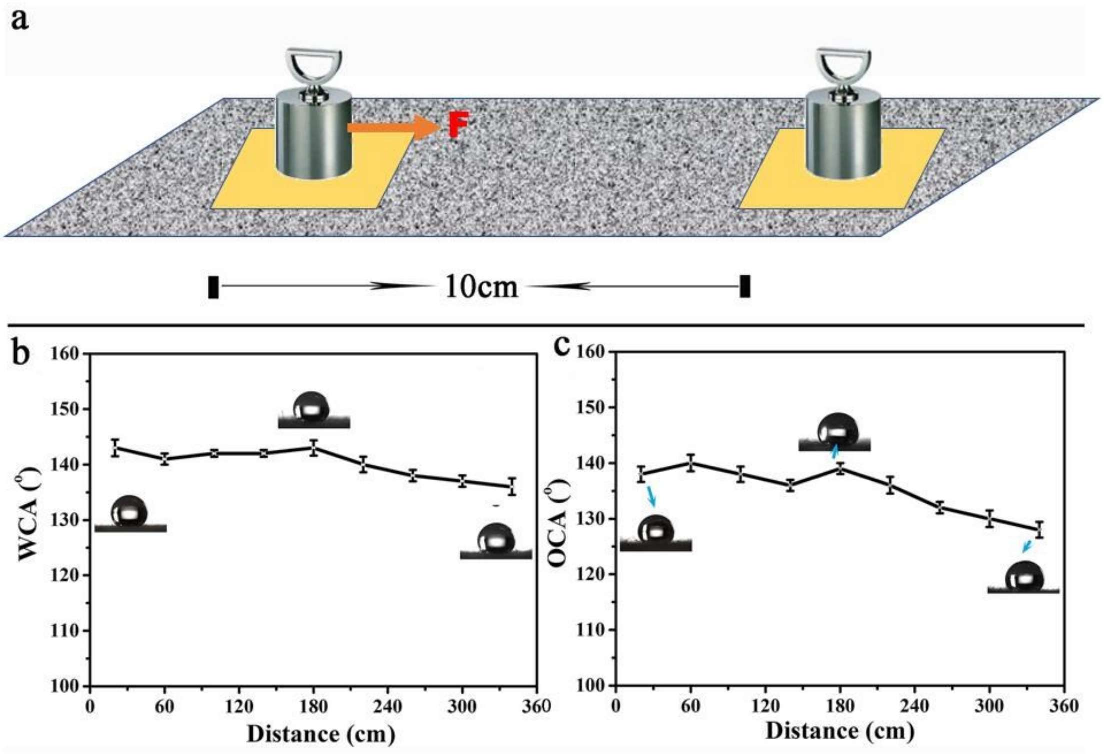

Figure S5 (a) The illustration for mechanical wear test. The WCA (b) and OCA (c) of PAR_7 after different wear length. The PAR_7 surface of was loaded with 294 N (normal direction) and then grind it horizontally with sandpaper (1500 meshes).

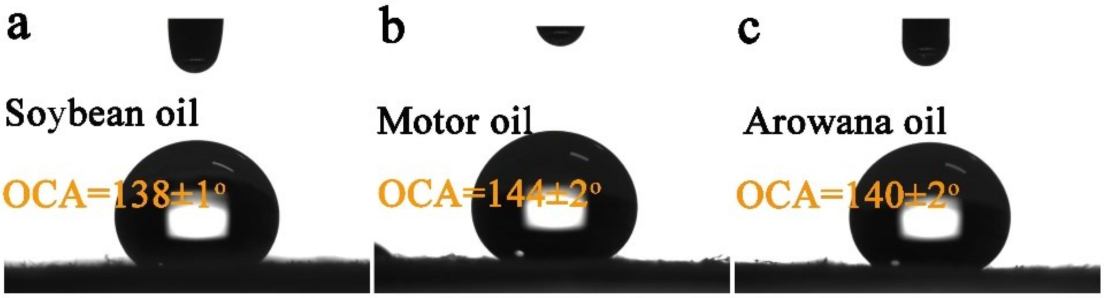

Figure S6 The OCA of soybean oil (a), motorcycle oil (b), and arowana oil (c) on the surface of PAR_7.

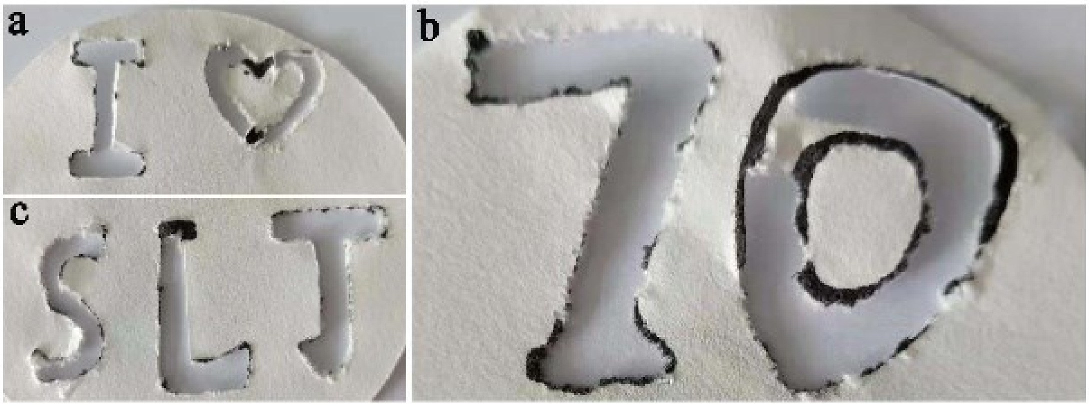

Figure S7 Homemade simple mold: (a) I♡-shape; (b) 70-shape; (c) SLJ-shape.
